# Circulating proteins in response to combined-modality therapy in rectal cancer identified by antibody array screening

**DOI:** 10.1186/s12885-016-2601-x

**Published:** 2016-07-26

**Authors:** Erta Kalanxhi, Helga Helseth Hektoen, Sebastian Meltzer, Svein Dueland, Kjersti Flatmark, Anne Hansen Ree

**Affiliations:** 1 Department of Oncology, Akershus University Hospital, Lørenskog, Norway; 2 Institute of Clinical Molecular Biology, Akershus University Hospital, P.O. Box 1000, 1478 Lørenskog, Norway; 3 Institute of Clinical Medicine, University of Oslo, P.O. Box 1171, Blindern, 0318 Oslo Norway; 4 Department of Tumor Biology, Oslo University Hospital – Norwegian Radium Hospital, Oslo, Norway; 5 Department of Oncology, Oslo University Hospital – Norwegian Radium Hospital, Oslo, Norway; 6 Department of Gastroenterological Surgery, Oslo University Hospital – Norwegian Radium Hospital, P.O. Box 4950, Nydalen, 0424 Oslo Norway

**Keywords:** Rectal cancer, Chemotherapy, Radiotherapy, Protein array, Serum proteins, Outcome

## Abstract

**Background:**

The increasingly complex programs of contemporary cancer therapy emphasize the need for biological indicators of both therapeutic response and adverse effects. One example is combined-modality treatment aimed at improving long-term outcome in patients with locally advanced rectal cancer, which commonly comes at the price of extended limits of patient tolerance.

**Methods:**

In a prospective study with intensified neoadjuvant treatment of rectal cancer patients, using an antibody array, the profiling of approximately 500 proteins was performed in serial serum samples collected at different stages of the treatment course.

**Results:**

The small number of proteins whose levels significantly changed after induction neoadjuvant chemotherapy (NACT) expanded substantially following the sequential chemoradiotherapy (CRT) and persisted four weeks later at treatment evaluation before pelvic surgery. Serum levels of proteins selected for validation of the experimental design, lipocalin-2 and matrix metalloproteinase-9, declined after NACT and gradually reverted to baseline values during the remaining neoadjuvant course. Of note, the greater the decline in post-NACT and post-CRT matrix metalloproteinase-9 levels, the more favorable progression-free survival. No correlation was found, however, with diarrhea scores, the clinical correlate of adverse therapeutic effects.

**Conclusions:**

Even though the findings were indicative of only tumor and not normal tissue effects, multiplex immunoassay analysis of circulating proteins in patients undergoing combined-modality therapy may in principle dissect the contribution of the individual modalities to overall systemic responses in patient outcome and tolerance.

**Trial registration:**

ClinicalTrials.gov NCT00278694; registration date: January 16, 2006, retrospective to enrollment of the first 10 patients of the current report.

**Electronic supplementary material:**

The online version of this article (doi:10.1186/s12885-016-2601-x) contains supplementary material, which is available to authorized users.

## Background

Colorectal cancer is characterized by heterogeneity at the molecular level and a complex tumor microenvironment [1]. Not surprisingly, patients may display very different responses to therapy, and treatment adapted to molecular markers has not always led to the expected outcome [2]. Moreover, the functional network of immune factors and the location and density of immune cells within the tumor microenvironment also contribute to the final clinical outcome [3, 4].

In treatment of locally advanced rectal cancer (LARC) with standard chemoradiotherapy (CRT) followed by surgery, local recurrence rates are low but metastatic disease remains a dominant cause of failure. The attempt of improving survival outcome by intensifying multimodal treatment protocols often extends towards the limits of normal tissue tolerance [5]. Prediction markers of treatment efficacy and toxicity might therefore assist in achieving more individualized treatment in LARC.

Multiplex analysis of protein patterns in tumors and systemically is one way of questing potential biomarkers [6]. Conceptually, detection of circulating proteins represents a negligibly invasive procedure by which the changing microenvironment within the tumor and its systemic manifestations, as well as the constitutional and acquired physiology of the patient, can be monitored in real-time. As such, correlation of proteins released into the circulation before, during, and after treatment with clinical parameters could enable the identification of biological indicators of treatment response and adverse effects.

**Fig. 1:**
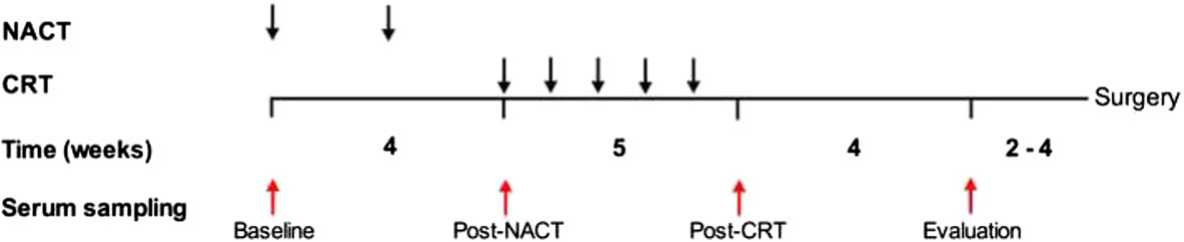
The timing of blood sampling (red arrows) within the treatment protocol. Black arrows indicate the start of each cycle of induction neoadjuvant chemotherapy (NACT) and of each consecutive week of the sequential chemoradiotherapy (CRT). A study-specific evaluation was undertaken before surgery, which was accomplished when the patient had recovered from the neoadjuvant therapy (commonly 2–4 weeks after evaluation)

Here, we have employed a commercially available antibody array technology to monitor serum levels of approximately 500 proteins in LARC patients within the context of a prospective study with an intensified neoadjuvant treatment schedule [7]. In this, the patients received induction neoadjuvant chemotherapy (NACT) and sequential CRT [8]. Serial serum samples were collected at baseline, following NACT (post-NACT), at CRT completion (post-CRT), and at evaluation of the neoadjuvant treatment four weeks thereafter (Fig. 1). The collection of proteins detected by the array analysis included immune factors, epithelial and vascular growth factors, and proteinases among others, which were anticipated to reflect proteins being released into the circulation from the tumor as well as from other tissues as an adverse response to treatment. Serum protein levels, which were technically validated by single-parameter analysis (enzyme-linked immunosorbent assay (ELISA) measurements) of the tumor microenvironmental factors lipocalin-2 (LCN2) and matrix metalloproteinase-9 (MMP9) [9, 10], were further correlated to progression-free survival (PFS) and treatment toxicities as prospectively assessed by Common Terminology Criteria for Adverse Events (CTCAE) scoring. Even though the present report showed an association between circulating MMP9 and treatment outcome but not tolerance, and it cannot be regarded as anything more than a pilot study, such a protein array analysis of serial serum samples may represent a rational approach for obtaining more global insight into systemic responses to increasingly complex programs of combined-modality radiotherapy.

## Methods

### Patients and treatment

The patient population within the current report, which is based on a prospective therapy study with five years of patient follow-up, was enrolled from October 5, 2005 through March 3, 2010. Patient eligibility criteria, evaluation procedures, and review procedures of follow-up have been detailed previously [8]. Because of the relatively low number of cases in the current set of analyses (*n* = 66 or lower, depending on the specific analysis; Additional file 1: Table S1), risk-adapted stratification of patients based on detailed tumor characteristics available from magnetic resonance imaging was waived. As outlined in Fig. 1, the treatment protocol consisted of induction NACT in terms of four weeks of oxaliplatin-containing chemotherapy followed by CRT. Radiation was delivered in daily 2-Gy fractions five days per week over a 5-week period with concomitant oxaliplatin weekly and capecitabine on days of radiation. Formal recording of adverse events, using CTCAE version 3.0, was performed throughout the neoadjuvant course. Surgery was planned 6–8 weeks after completion of the neoadjuvant treatment. In accordance with national guidelines at the time, patients did not proceed to further therapy.

### Serum sampling

From the 66 study cases within the current report (Additional file 1: Table S1), serum had been collected at baseline (*n* = 66), post-NACT (*n* = 61), post-CRT (*n* = 59), and at the time of treatment evaluation (*n* = 55). The collection, processing, and storage of samples followed a standardized protocol, where blood was drawn in plain serum tubes with no additives for centrifugation to separate serum, which was left on ice for no more than one hour before storage at −80 °C. The antibody array analysis was undertaken in January 2013 (i.e., after 31–87 months of storage) and the ELISA analysis in November 2014 (i.e., after 53–109 months), all after only one freeze/thaw cycle of the samples.

### Antibody array technology and data analysis

Serum samples were analyzed with a high-density antibody array that was chosen for the coverage of stromal proteins such as immune factors, epithelial and vascular growth factors, and proteinases (AAH-BLG-1; RayBiotech Inc., Norcross, GA, USA) at the Genomics Core Facility, Oslo University Hospital, as detailed previously [5]. Briefly, proteins were biotinylated and added onto glass slides pre-printed with 507 capture antibodies. Bound proteins (in duplicates) were detected with a streptavidin-conjugated fluorescent dye, and the resulting array image spots were converted to numerical values. The array data is available in the Gene Expression Omnibus repository (GEO Accession Number GSE65622).

Following data processing as detailed in the supplementary information within the deposited data, the data was transformed to natural logarithms and Significance Analysis of Microarrays (SAM) software version 5.0 was used to determine alterations in protein levels during treatment by employing paired analysis and a false discovery rate cut-off of 10 % [11]. SAM, originally developed for analysis of DNA microarrays, can also be utilized for protein arrays. Herein, changes in protein levels are identified by a set of *t*-tests, where each protein receives a score on the basis of its change relative to the standard deviation of repeat measurements. This software handles any missing data by imputation using the *K*-nearest neighbor method [11]. Functional association analysis of the resulting list of altered proteins was performed by the Functional Coupling software version 3.0 [12] using a maximum of five nodes per expansion step and a confidence threshold of 0.5.

### ELISA analysis of LCN2 and MMP9

This was performed with the Quantikine® Human Lipocalin-2/NGAL and MMP9 Immunoassays (R&D Systems, Minneapolis, MN, USA) according to the manufacturer’s manuals. For this analysis, serum samples were diluted 1:20 and 1:200 for quantification of LCN2 and MMP9, respectively. All samples were analyzed in duplicates.

### Study endpoints

The clinical endpoints were treatment toxicities (CTCAE scores) during neoadjuvant therapy, histologic tumor response, and PFS. Follow-up data was censored on August 8, 2013. Within the context of the study [8], the resected tumor specimens were prepared in accordance with the requirements of a validated protocol [13] and histologically evaluated for treatment response according to standard staging (ypTN; TNM version 5). In this patient population of locally advanced tumors (mainly T3–4 cases), ypT0–2 outcome was considered as good response and correspondingly, ypT3–4 results were regarded as poor tumor shrinkage. Moreover, response in the resected specimen was graded within one of five tumor regression grade (TRG) categories [14], where TRG1 represents absence of residual tumor cells (pathologic complete response), TRG2 corresponds to the presence of sparsely remaining tumor cells scattered in fibrosis or mucin, TRG3 is an intermediate score between TRG2 and TRG4, the latter defining residual tumor that outgrows fibrosis or mucin, and TRG5 corresponds to the lack of morphologic signs of response. Of note, when responding to neoadjuvant treatment, LARC frequently shows fragmentation into microscopic residual disease [15]. Consequently, it is rational to group TRG2 together with TRG1 as good histologic regression and correspondingly, the range of TRG3–5 scores as poor response. In cases of ambiguity about the exact histologic tumor response of the surgical specimen, the meticulous procedure of targeted magnetic resonance-guided histopathology [15] was applied.

### Statistical analysis

Analyses were performed using IBM SPSS Statistics for Windows version 23.0 or GraphPad Prism version 6.0 h. Correlation between continuous data was determined by Pearson product correlation analysis or one-way analysis of ranks. Categorical data was compared by paired *t*-test. Estimated 5-year PFS was calculated from the time of study enrollment to the date of recurrent disease, death of any cause, or end of follow-up (five years after the date of surgery), whichever came first. Associations between selected variables and PFS were modeled with univariate Cox regression analysis. All tests were two-sided. A *p*-value less than 0.05 was considered statistically significant. Importantly, during array data processing, some fluorescence intensity measurements were filtered out, and that explains the differences between the total number of patients with available array data and the number of measurements for a particular protein (Additional file 1: Table S1).

## Results

### Circulating proteins during the course of neoadjuvant treatment

**Fig. 2:**
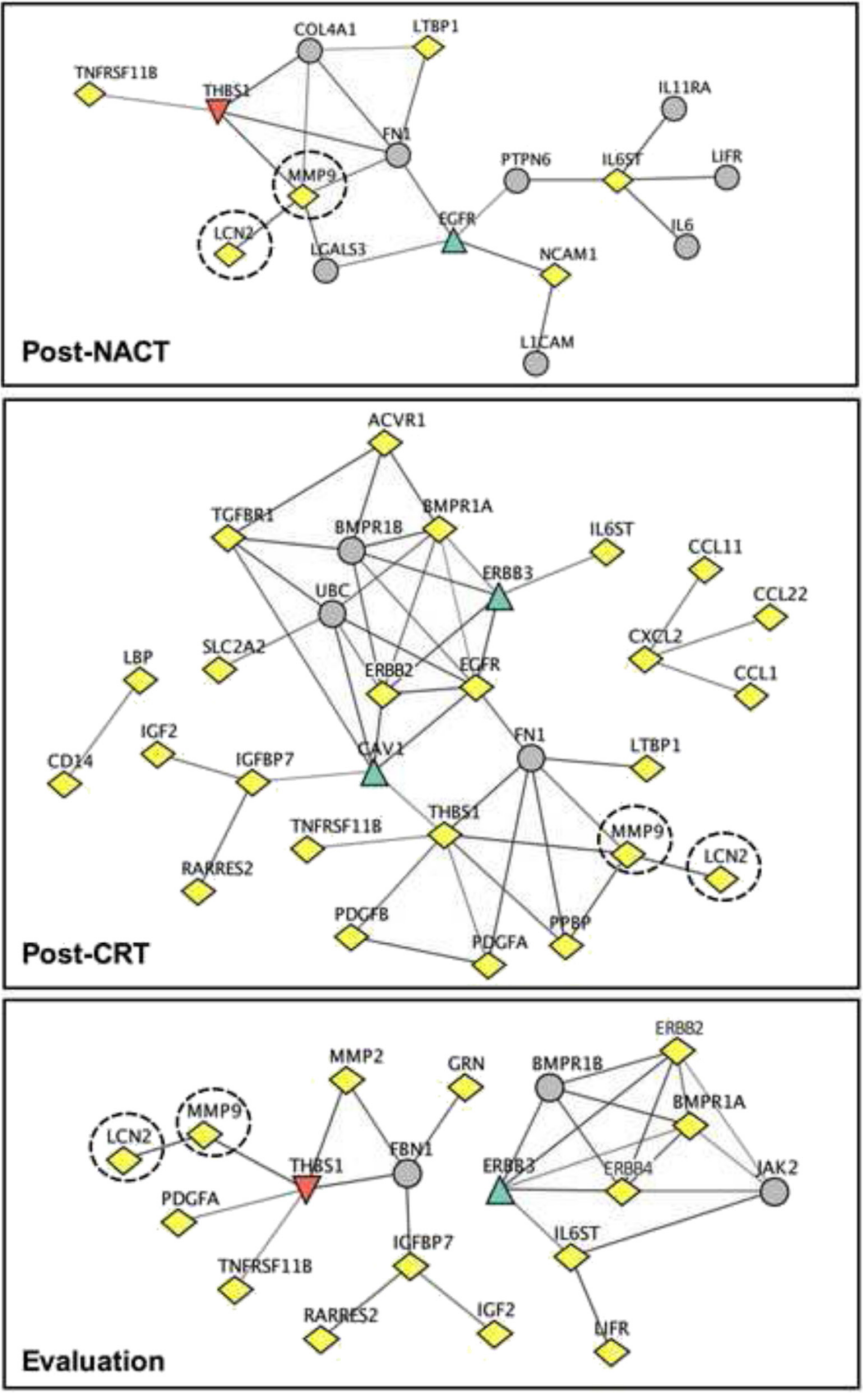
Functional coupling between proteins that changed in patients’ circulation during neoadjuvant therapy. Proteins are depicted by their gene symbols. Yellow nodes: proteins whose serum levels significantly differed from baseline following induction neoadjuvant chemotherapy (post-NACT) and sequential chemoradiotherapy (post-CRT) and at treatment evaluation. Non-yellow nodes: proteins not present in the query list but predicted as interacting with the significantly altered proteins at the specific sampling point. Encircled nodes: proteins further analyzed

Using the antibody array, approximately 500 proteins were measured in serial serum samples collected from the study patients at baseline, post-NACT, post-CRT, and at evaluation of the neoadjuvant treatment. Significant changes in protein levels (fold-change relative to baseline) were observed during the treatment course, but only for six proteins (ADIPOQ, ANG, IL6ST, LCN2, MMP9, and TNFRSF11B; identities given by the gene names) persistently at every sampling point and considerably higher numbers (38–46 proteins) at the post-CRT and evaluation sampling points (Table 1). Functional coupling analysis revealed a high degree of interaction between some of the altered proteins, and additionally, it predicted interactions with proteins that were not present in the query list (Fig. 2). One example, as can be seen from comparison of Table 1 and Fig. 2, was thrombospondin-1 (THBS1), which was not present in the list of proteins whose serum levels were altered by NACT but was predicted as a functional interaction partner of MMP9, one of the few proteins with significant change at this early stage of treatment. At CRT completion, however, the thrombospondin-1 levels were also significantly altered from baseline. This may indicate that the induction NACT initiated a biological response that was strongly enhanced by the sequential CRT.

**Table 1:** Significantly altered serum proteins in study patients

Post-NACT		Post-CRT				Evaluation			
*ADIPOQ*	1.15	ACVR1	1.10	IGFBP3	1.21	*ADIPOQ*	1.10	GRN	1.12
*ANG*	1.21	*ADIPOQ*	1.10	IGFBP7	1.12	*ANG*	1.11	IGF2	1.21
IGFBP2	1.15	*ANG*	1.21	IL1RAPL2	1.10	ANGPT2	1.16	IGFBP7	1.15
*IL6ST*	1.12	BMPR1A	1.11	IL27	1.12	BDNF	1.11	IL1RAPL2	1.11
NCAM1	1.12	CCL1	1.11	*IL6ST*	1.17	BMPR1A	1.18	IL22	1.12
SAA1	1.15	CCL11	1.12	LBP	1.16	CCL11	1.14	*IL6ST*	1.12
*TNFRSF11B*	1.34	CCL22	1.13	LEPR	1.11	CCR6	1.14	LIFR	1.12
		CCR6	1.10	PLAU	1.12	CD14	1.13	MMP2	1.10
		CD14	1.14	RARRES2	1.12	CSF1	1.19	NGFB	1.12
		CSF1	1.22	RELT	1.11	CTF1	1.12	NTF4	1.13
		EGFR	1.11	SAA1	1.27	CXCR1	1.19	RARRES2	1.23
		ERBB2	1.20	SIGLEC5	1.14	CXCR5	1.11	SIGLEC9	1.12
		FLT3LG	1.11	SIGLEC9	1.13	CXCR6	1.12	SLC2A2	1.19
		GCG	1.13	SLC2A2	1.13	ERBB2	1.11	THBS4	1.12
		GRN	1.16	TGFBR1	1.11	ERBB4	1.11	*TNFRSF11B*	1.16
		IGF2	1.18	THBS4	1.10	FLT3LG	1.12		
		IGFBP2	1.15	*TNFRSF11B*	1.65				
*LCN2*	0.65	CHRDL2	0.88	PDGFA	0.86	CHRDL2	0.86	PDGFA	0.88
LTBP1	0.87	CXCL2	0.88	PDGFB	0.89	FGF13	0.84	S100A12	0.84
*MMP9*	0.63	FGF13	0.83	PF4	0.85	*LCN2*	0.84	TMEFF2	0.89
		*LCN2*	0.73	PPBP	0.83	*MMP9*	0.74		
		LTBP1	0.80	S100A12	0.85				
		*MMP9*	0.68	**THBS1**	0.84				

Using the Significance Analysis of Microarrays software, serum protein levels (entered as antibody array fluorescence intensities transformed to natural logarithms) that were significantly altered from baseline following induction neoadjuvant chemotherapy (post-NACT) and sequential chemoradiotherapy (post-CRT) and at evaluation of the neoadjuvant treatment were determined. The fold-change increase or decrease from baseline is indicated to the right of each protein. False discovery rate was less that 10 % for all proteins. Proteins are listed by their gene names. Proteins with serum levels that were significantly different from baseline at every other sampling point are italicized. The protein highlighted in bold is also discussed in the current report. The crude table has been shown in a previous report [18]

### Validation of antibody array measurements

**Fig. 3:**
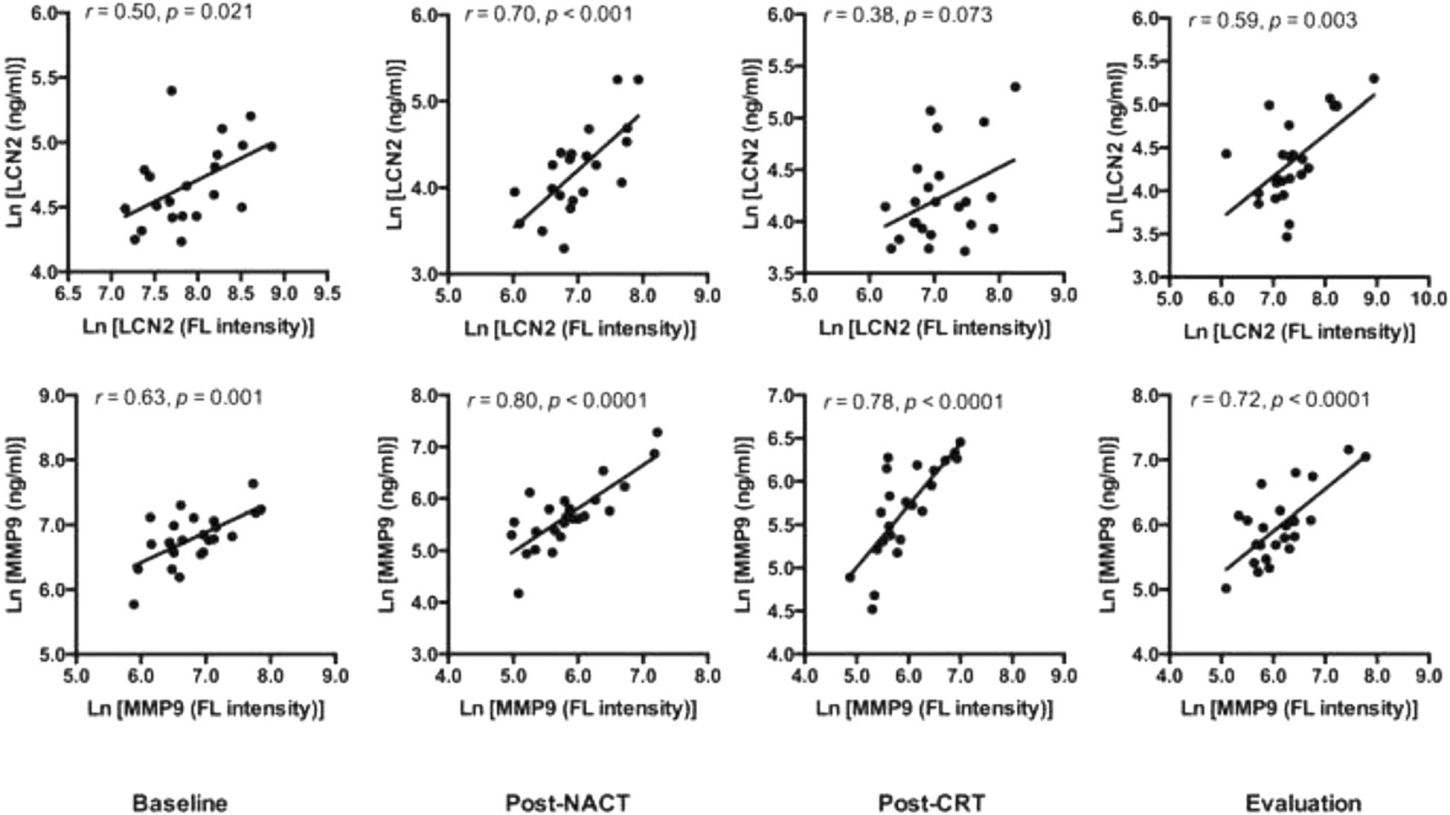
Correlations between array fluorescence (FL) intensities and single-parameter immunoassay measurements. Values (transformed to natural logarithms) of lipocalin-2 (LCN2) and matrix metalloproteinase-9 (MMP9) levels in serum samples obtained at baseline, following induction neoadjuvant chemotherapy (post-NACT) and sequential chemoradiotherapy (post-CRT), and at treatment evaluation from 24 randomly chosen patients were compared

This was achieved by ELISA measurements of LCN2 and MMP9, being two of the six proteins that were altered at every sampling point (Table 1). Another reason for choosing these proteins for this purpose was that LCN2 and MMP9 form a covalent complex [16], as reflected in Fig. 2, and their regulatory role in tumor microenvironmental biology [17]. In serum samples from 24 randomly chosen patients, significant correlation between array measurements (in fluorescence intensities) and serum protein levels (in ng/ml) was found for both LCN2 and MMP9 at all sampling points with exception of LCN2 post-CRT (Fig. 3).

Three of the four serum proteins that had significantly increased array values from baseline at every subsequent sampling point (ADIPOQ, ANG, and IL6ST; Table 1) were not analyzed by alternative methods. The fourth of the elevated proteins, the soluble tumor necrosis factor decoy receptor TNFRSF11B, was thoroughly investigated in a separate study which was recently reported [18]. In neither case, altered array values were associated with long-term patient outcome (Additional file 1: Table S2).

### Circulating LCN2 and MMP9 during neoadjuvant treatment

**Fig. 4:**
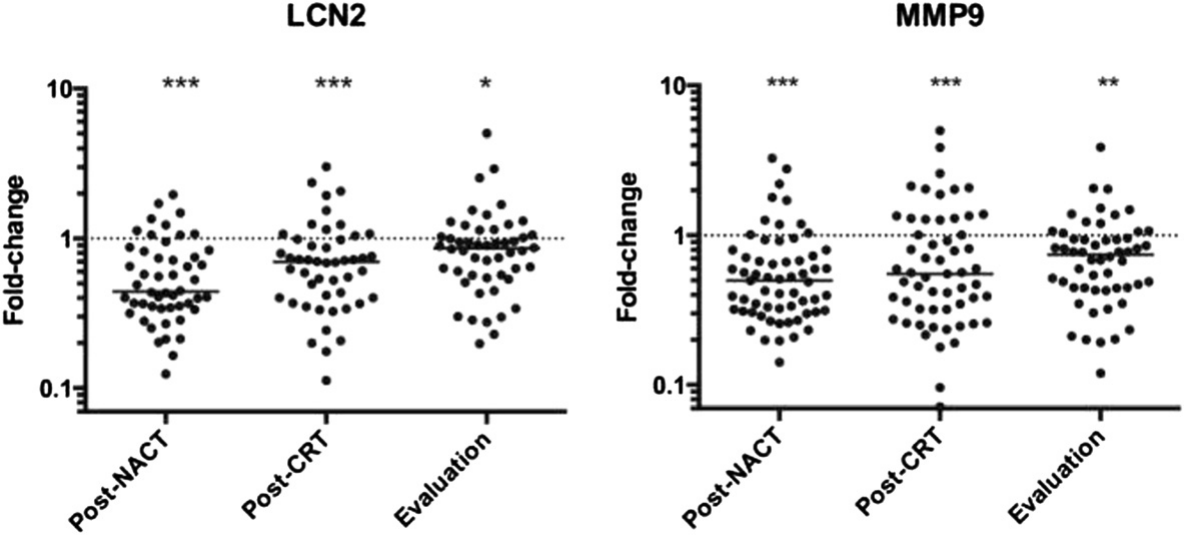
Serum lipocalin-2 (LCN2) and matrix metalloproteinase-9 (MMP9) levels during neoadjuvant therapy. Array fluorescence intensities relative to the individual patient’s baseline values following induction neoadjuvant chemotherapy (post-NACT; *n* = 50 for LCN2 and *n* = 61 for MMP9) and sequential chemoradiotherapy (post-CRT; *n* = 48 for LCN2 and *n* = 57 for MMP9) and at treatment evaluation (*n* =50 for LCN2 and *n* =54 for MMP9); lines, median group values; *p < 0.01; **p < 0.001; ***p < 0.0001

Figure 4 illustrates serum levels of LCN2 and MMP9 as determined by the antibody array. For both proteins, the group values at all of the sampling points were significantly lower than at baseline, with [median (range) values given for LCN2 and MMP9, respectively] a post-NACT fold-change decline to 0.44 (0.12–2.0) and 0.49 (0.14–3.3) followed by a gradual increase to 0.69 (0.11–3.0) and 0.55 (0.07–5.0) post-CRT. At evaluation a few weeks before surgery, LCN2 and MMP9 values had further approached baseline with fold-changes of 0.86 (0.20–5.0) and 0.75 (0.12–3.9).

### Circulating LCN2 and MMP9 and disease outcome

Since both the post-NACT and post-CRT sampling points immediately followed completion of a defined therapeutic modality, we investigated whether the individual patient’s decline in serum levels might predict response to the combined-modality therapy. No correlations were seen for ypTN stage or TRG score (Additional file 1: Table S3). When last censored, median follow-up time for patients with the relevant paired LCN2 and MMP9 measurements was 63 months (range 3–65). Three patients had experienced local recurrence as the first event of disease relapse. In addition, 13 and 11 cases in the LCN2 and MMP9 groups, respectively, had metastatic progression as the first event. Hence, PFS was chosen as the relevant long-term endpoint.

On univariate Cox regression analysis (Table 2), histologic treatment response was highly associated with clinical outcome, underpinned by correlation of both ypT3–4 and ypN1–2 stages as well as TRG 3–5 scores with adverse PFS. Moreover, the lower the post-NACT and post-CRT MMP9 values (fold-change decline from baseline), the more favorable PFS. With increasing MMP9 values (i.e., less decline from baseline), the hazard ratio for a PFS event was almost 3. For LCN2, the impact of post-NACT and post-CRT changes on PFS just failed to reach significance. Multivariate analysis was waived because of the low number of PFS events. Differences in PFS between patients separated into groups based on the magnitude of the serum LCN2 and MMP9 changes are visualized in Additional file 2: Figure S1.

**Table 2:** Progression-free survival – univariate analysis

		HR	95 % CI	*p*-value
Age		1.0	0.95–1.9	0.12
TN stage	T2–3			
	T4	1.9	1.0–3.9	0.06
	N0			
	N1–2	0.97	0.38–2.5	0.95
Baseline CEA	≤ULN			
	>ULN	1.4	0.72–2.8	0.31
ypTN stage	ypT0–2			
	ypT3–4	4.2	1.9–9.5	<0.001
	ypN0			
	ypN1–2	3.7	1.8–7.5	<0.001
TRG score	TRG1–2			
	TRG3–5	2.4	1.2–4.7	0.01
Post-NACT	LCN2	2.2	1.0–4.8	0.05
	MMP9	2.8	1.5–5.9	<0.01
Post-CRT	LCN2	2.3	1.0–5.2	0.06
	MMP9	2.9	1.1–3.4	0.03

Adjusted hazard ratio (HR) with 95 % confidence interval (CI); HR above 1: higher probability of unfavorable progression-free survival for the higher value(s) of the parameter. Age and altered serum values (array fluorescence intensities) as fold-change from baseline of lipocalin-2 (LCN2) and matrix metalloproteinase-9 (MMP9) after induction neoadjuvant chemotherapy (post-NACT) and sequential chemoradiotherapy (post-CRT): entered as continuous data from lowest to highest measurements. *CEA* carcinoembryonic antigen, *ULN* upper limit of normal, *TRG* tumor regression grade

### Circulating LCN2 and MMP9 and treatment toxicities

Neither LCN2 nor MMP9 shows tumor-specific expression, and the release of these proteins into the circulation from other tissues of origin might therefore change as an adverse response to therapy. Because the present study regimen caused intestinal toxicity [8], we investigated the possibility that the extent of decline in serum LCN2 and MMP9 levels might also, in susceptible individuals, predict treatment-induced enteropathy, clinically presenting as diarrhea.

This analysis omitted patients reporting diarrhea at baseline as a presenting symptom of their disease or from whom definite maximum CTCAE score was not available. The remaining cases (Additional file 1: Table S1) were grouped according to maximum CTCAE scores (from 0 to 3; no case of CTCAE grade 4 diarrhea was reported). As could be expected, the scores recorded at CRT completion distinguished patients with respect to intestinal toxicity, consistent with radiation-induced acute toxicity being cumulative. While group 0 consisted of cases devoid of diarrhea (i.e., CTCAE grade 0) throughout the treatment course, group 1, 2, and 3 patients reported CTCAE grade 1, 2, and 3 post-CRT diarrhea, respectively. With this categorization, using post-NACT and post-CRT values of LCN2 and MMP9 (fold-change from baseline) from cases that provided these particular measurements (*n* = 38–49), no correlation was found with this specific adverse treatment event (Additional file 1: Table S4).

Moreover, because LCN2 is expressed in complex with MMP9 in neutrophils specifically [9], we asked whether the decline in serum levels might have reflected grave neutropenia during NACT and CRT. However, only one CTCAE grade 3 (and none grade 4) neutropenic event was reported by the total population of 66 patients. Still, the individual patient’s absolute neutrophil count at completion of the systemic modality (NACT) showed moderate correlation with both LCN2 and MMP9 values (fold-change from baseline; LCN2: *r* = 0.35, *p* = 0.015; MMP9: *r* = 0.44, *p* = 0.001). Other CTCAE grade 3–4 events recorded in the study population [8] were also infrequent and therefore deemed unsuitable for analysis in the current study subpopulation.

## Discussion

The essential idea of this study was to monitor circulating proteins in LARC patients receiving induction NACT and sequential CRT using a multiplex antibody array in an effort to quest readily accessible indicators of treatment efficacy and toxicity to combined-modality radiotherapy. Protein profiles from serial serum samples collected at different stages of the treatment course were related to PFS (patient outcome) and CTCAE scores (patient tolerance), and the strategy was corroborated by examining LCN2 and MMP9 specifically.

There are several advantages of using the antibody array technology in the context of this study. In addition to proteins being potentially targetable within a combined-modality treatment protocol [7], their expression integrates biological responses at transcription, post-translational modification, and compartmentalization, including secretion. Importantly, simultaneous analysis of a manifold of proteins as compared to the investigation of single markers gives a more complete picture of systemic patient responses during the treatment course. However, high-throughput technologies commonly come with certain limitations. Detection of only half a thousand out of an abundance of proteins released into the serum compartment represents an investigation bias. In addition, there are technical aspects such as protein stability and non-specific binding, which could result in low detection of some proteins or generation of false positives, respectively [19].

Since analytical pipelines for protein arrays are still at their infancy, we adopted methods developed for genomic data to place the findings into a biological context. The array data revealed that only a few serum proteins changed during the induction NACT but that the sequential CRT modality affected a substantial number. At the time of treatment evaluation four weeks after CRT completion, a major part of these protein alterations were still present. Exploration of possible functional networks of the altered proteins indicated an emerging systemic response elicited by the initial NACT which was considerably amplified by the CRT, illustrated by the detected immune factors, growth factors, and proteinases. Importantly, the use of this technology for investigation of circulating biomarkers within the context of a prospective study represents a negligibly invasive approach that may bring insights into the patients’ systemic responses with regard to both tumor and normal tissue effects to the various treatment modalities.

Since the antibody array at best is semi-quantitative, any in-depth investigation of potential markers of treatment response or adverse effects would require validation by a supplementary methodology. We chose two proteins for single-parameter ELISA analysis – LCN2 and MMP9, and their serum levels in ng/ml showed significant correlation with the array measurements in fluorescence intensities. Levels of both factors declined significantly following the induction NACT, then gradually increased during the sequential CRT, and finally reverted close to baseline at the evaluation sampling point. MMP9 is involved in extracellular matrix remodeling [10] and LCN2, which is predominantly produced by neutrophils, forms a covalent complex with MMP9 and protects it from auto-degradation [9, 16, 20, 21]. Enhanced tissue expression as well as circulating levels of LCN2 and MMP9, or their complex, have been observed in several tumor entities [9, 22–25]. In a study of primarily resected colorectal cancers, primary tumor MMP9 expression was an independent predictor of disease-free survival [26]. Baseline tumor biopsies in our study, which were sampled from the endoluminal surface of the deeply invasive tumors, would probably not have enabled a reliable estimation of whole tumor MMP9 expression for direct comparison with the patients’ circulating MMP9 levels. Nevertheless, baseline serum levels of LCN2 and MMP9 (as determined by the ELISA measurements) in 24 cases were similar to previously reported values in patients with colorectal cancer [27–29] and considerably higher than those in healthy subjects [22]. Distinct declines in serum MMP9 levels following the course of treatment initiation and sequential CRT were associated with advantageous PFS. It is tempting to speculate that NACT, as induction chemotherapy, and aided by the CRT, may specifically have targeted tumor microenvironmental components with MMP9-regulated inflammatory and metastatic properties [17] in good-prognosis patients.

Importantly, we could not exclude the possibility that alterations in serum contents of LCN2 and MMP9 reflected deleterious normal tissue effects. With the particular study design, the pelvic CRT caused intestinal toxicity [8]. Moreover, any associations between circulating LCN2 and MMP9 levels and neutrophil counts [9] during chemotherapy should be considered. In other words, both the normal bowel and neutrophils locally or systemically might contribute to alterations in circulating levels of these proteins. However, there was no correlation between the decline in serum levels of LCN2 and MMP9 and the diarrhea score. Essentially, the study displayed low incidence of CTCAE grade 3–4 adverse effects, probably reflecting the precaution criteria in the study protocol. In this, the neoadjuvant treatment schedule was continuously adjusted according to toxicity by reducing doses of or entirely discontinuing oxaliplatin, capecitabine, or radiotherapy in that order of priority, in accordance with the relative importance of the three therapeutic components within the study regimen. These safety concerns may have precluded the quest for circulating markers of treatment toxicity.

Of note, the number of study cases from whom serum samples were available was small (from 66 at baseline to 55 before surgery), although a tentative sample size analysis using 5-year PFS rates as estimated in Additional file 2: Figure S1 indicated that the numbers of post-NACT LCN2 and MMP9 cases and post-CRT MMP9 cases were sufficient for the purpose. Nevertheless, the study findings should ideally have been evaluated in an independent patient cohort where cases had undergone combined-modality radiotherapy with curative intent for a pelvic malignancy along with the simultaneous prospective serial sampling of serum (or plasma) throughout the treatment course and where additionally, results from prospective recording of adverse events and long-term outcome data were available. Our attempts into identifying such validation material have not been successful.

## Conclusions

To summarize, this report is based on a prospective study of an intensified neoadjuvant therapy regimen in LARC patients with median follow-up time of five years after definitive surgery. The study population of mainly T3–4 cases was treated with curative intent but had metastatic progression as a main PFS event, and the recorded spectrum of CTCAE grade 0–3 diarrhea scores reflected the range of individual adverse responses to the therapy. As a conceptual approach to delineate possible mechanisms involved in tumor and systemic responses to the combined-modality radiotherapy, we employed a high-density antibody array technology to monitor changes in patients’ serum protein levels during the treatment course. The experimental strategy was illustrated by further investigation of selected proteins, and specifically, treatment-induced changes in circulating MMP9 levels were associated with PFS but not treatment toxicity. However, the current study had several limitations specifically with regard to a rather low patient number, and further studies with larger populations are required to validate the overall design as well as the specific findings.

Essentially, in order to improve long-term outcome of advanced cancer disease, increasingly complex contemporary therapies raise the requirement for biological indicators of treatment efficacy and toxicity (i.e., patient outcome and tolerance). In this context, the use of multiplex immunoassay technology to monitor circulating proteins in patients undergoing a combined-modality treatment program may represent a promising path to treatment-related biomarker discovery. Analysis of several markers simultaneously, as opposed to single candidates, may enlighten the complex biological responses that are elicited, and the detection of alterations in protein levels at different stages of the treatment course may help in dissecting the contribution of the single modalities to the overall treatment response or adverse effects.

## Abbreviations

CRT, chemoradiotherapy; CTCAE, Common Terminology Criteria for Adverse Events; ELISA, enzyme-linked immunosorbent assay; LARC, locally advanced rectal cancer; LCN2, lipocalin-2; MMP9, matrix metalloproteinase-9; NACT, neoadjuvant chemotherapy; PFS, progression-free survival; SAM, Significance Analysis of Microarrays; TRG, tumor regression grade

## Additional files

Additional file 1: Table S1. Numbers of serum samples from study patients. The initial population of 66 study patients had serum sampled at baseline, following induction neoadjuvant chemotherapy (post-NACT) and sequential chemoradiotherapy (post-CRT), and at evaluation of the neoadjuvant treatment. Applying Common Terminology Criteria for Adverse Events (CTCAE) version 3.0 and omitting cases reporting CTCAE grade >0 diarrhea at baseline or from whom definite maximum CTCAE score was not available, the initial number of 53 study patients remained for the analysis of treatment toxicity (maximum diarrhea CTCAE score) during the neoadjuvant treatment course. Missing values as a result of processing of the antibody array data from serum samples led to reduction in the number of cases with lipocalin-2 (LCN2) and matrix metalloproteinase-9 (MMP9) measurements and subsequently, the number of cases with matched values for fold-change from baseline ratios available for down-stream analyses. **Table S2.** Association between altered serum values (array fluorescence intensities as fold-change from baseline) of the four listed proteins (identities given by the gene names) at completion of induction neoadjuvant chemotherapy (post-NACT) and sequential chemoradiotherapy (post-CRT), entered as continuous data from lowest to highest measurements, and PFS was modeled by Cox regression analysis and given as adjusted hazard ratio (HR) with 95% confidence interval (CI); HR above 1: higher probability of unfavorable PFS for the higher value(s) of the parameter. In neither case, altered array values at completion of NACT or CRT were associated with PFS. **Table S3.** Study patients’ characteristics. The initial population of 66 study patients had serum sampled at baseline, following induction neoadjuvant chemotherapy (post-NACT) and sequential chemoradiotherapy (post-CRT), and at evaluation of the neoadjuvant treatment. One patient had disease progression in the pelvic cavity during the neoadjuvant treatment and therefore proceeded to palliative surgery. As a consequence, histologic tumor response data (ypTN stage and tumor regression grade (TRG) scoring) were missing. The serum samples were analyzed with an antibody array. Array data processing led to reduction in the number of cases with lipocalin-2 (LCN2) and matrix metalloproteinase-9 (MMP9) measurements and subsequently, the number of cases with matched values for fold-change from baseline ratios – given here – that were available for down-stream analyses. Distribution of parameters was analyzed using Pearson product correlation for continuous data and paired *t*-test for categorical variables. Within the total study population as well as all of the LCN2 and MMP9 groups of study cases, patients’ characteristics were equally distributed. **Table S4.** Treatment-induced enteropathy in study patients. Using one-way analysis of ranks, altered serum values (array fluorescence intensities as fold-change from baseline) of lipocalin-2 (LCN2) and matrix metalloproteinase-9 (MMP9) following induction neoadjuvant chemotherapy (post-NACT) and sequential chemoradiotherapy (post-CRT) were compared for patients categorized into four groups of maximum diarrhea scores, as assessed by Common Terminology Criteria for Adverse Events (CTCAE) version 3.0, during the neoadjuvant treatment course. Categories are Group 0: CTCAE grade 0 diarrhea; Group 1: CTCAE grade 1 diarrhea; Group 2: CTCAE grade 2 diarrhea; Group 3: CTCAE grade 3 diarrhea. (DOC 99 kb) Additional file 2: Figure S1. Alterations in serum levels of lipocalin-2 (LCN2; left panel) and matrix metalloproteinase-9 (MMP9; right panel) – progression-free survival (PFS). Patients were categorized into groups according to the magnitude of decline in serum values (array fluorescence intensities as fold-change from baseline) at completion of induction neoadjuvant chemotherapy which resulted in a value either below (solid line) or above (dashed line) a calculated optimum threshold (by receiver-operating characteristic analysis) for favorable PFS. Crude differences in PFS were assessed by the log-rank test and visualized by the Kaplan-Meier method. Patients with fold-change values below threshold (i.e., more decline from baseline) during the chemotherapy had significantly better 5-year PFS than those with fold-change values above threshold (i.e., less decline from baseline); for LCN2: 76% versus 38% (*p* = 0.004, *n* = 50, threshold of 0.8); for MMP9: 76% versus 20% (*p* = 0.0002, *n* = 61, threshold of 1.0). The corresponding identification of optimum threshold values for LCN2 and MMP9 alterations at completion of the sequential chemoradiotherapy was not possible with the actual data sets. (DOC 1827 kb)
