# Supplementary material for: Circulating proteins in response to combined-modality therapy in rectal cancer identified by antibody array screening
Source: BMC Cancer. 2016 Jul 26;16:536. doi: 10.1186/s12885-016-2601-x (PMC4962367; doi:10.1186/s12885-016-2601-x)
Supplement: Additional file 1: Table S1. — Numbers of serum samples from study patients. The initial population of 66 study patients had serum sampled at baseline, following induction neoadjuvant chemotherapy (post-NACT) and sequential chemoradiotherapy (post-CRT), and at evaluation of the neoadjuvant treatment. Applying Common Terminology Criteria for Adverse Events (CTCAE) version 3.0 and omitting cases reporting CTCAE grade >0 diarrhea at baseline or from whom definite maximum CTCAE score was not available, the initial number of 53 study patients remained for the analysis of treatment toxicity (maximum diarrhea CTCAE score) during the neoadjuvant treatment course. Missing values as a result of processing of the antibody array data from serum samples led to reduction in the number of cases with lipocalin-2 (LCN2) and matrix metalloproteinase-9 (MMP9) measurements and subsequently, the number of cases with matched values for fold-change from baseline ratios available for down-stream analyses. Table S2. Association between altered serum values (array fluorescence intensities as fold-change from baseline) of the four listed proteins (identities given by the gene names) at completion of induction neoadjuvant chemotherapy (post-NACT) and sequential chemoradiotherapy (post-CRT), entered as continuous data from lowest to highest measurements, and PFS was modeled by Cox regression analysis and given as adjusted hazard ratio (HR) with 95% confidence interval (CI); HR above 1: higher probability of unfavorable PFS for the higher value(s) of the parameter. In neither case, altered array values at completion of NACT or CRT were associated with PFS. Table S3. Study patients’ characteristics. The initial population of 66 study patients had serum sampled at baseline, following induction neoadjuvant chemotherapy (post-NACT) and sequential chemoradiotherapy (post-CRT), and at evaluation of the neoadjuvant treatment. One patient had disease progression in the pelvic cavity during the neoadjuvant treatment and therefore proc [file 12885_2016_2601_MOESM1_ESM.doc]

**Additional file 1**

**Table S1** Numbers of serum samples from study patients

|  | **From the entire study population** | | |  | **From patients with CTCAE grade 0 diarrhea at baseline and available maximum CTCAE diarrhea score** | | |
| --- | --- | --- | --- | --- | --- | --- | --- |
|  | Available samples | Samples with protein array values | Samples with array fold-change values |  | Available samples | Samples with protein array values | Samples with array fold-change values |
|  | *n* | *n* | *n* |  | *n* | *n* | *n* |
| **Baseline** | 66 |  |  |  | 53 |  |  |
| LCN2 |  | 61 |  |  |  | 48 |  |
| MMP9 |  | 65 |  |  |  | 53 |  |
| **Post-NACT** | 61 |  |  |  | 49 |  |  |
| LCN2 |  | 53 | 50 |  |  | 43 | 40 |
| MMP9 |  | 61 | 61 |  |  | 49 | 49 |
| **Post-CRT** | 59 |  |  |  | 48 |  |  |
| LCN2 |  | 53 | 48 |  |  | 43 | 38 |
| MMP9 |  | 58 | 57 |  |  | 48 | 48 |
| **Evaluation** | 55 |  |  |  | 44 |  |  |
| LCN2 |  | 52 | 50 |  |  | 42 |  |
| MMP9 |  | 55 | 54 |  |  | 44 |  |

The initial population of 66 study patients had serum sampled at baseline, following induction neoadjuvant chemotherapy (post-NACT) and sequential chemoradiotherapy (post-CRT), and at evaluation of the neoadjuvant treatment. Applying Common Terminology Criteria for Adverse Events (CTCAE) version 3.0 and omitting cases reporting CTCAE grade >0 diarrhea at baseline or from whom definite maximum CTCAE score was not available, the initial number of 53 study patients remained for the analysis of treatment toxicity (maximum diarrhea CTCAE score) during the neoadjuvant treatment course. Missing values as a result of processing of the antibody array data from serum samples led to reduction in the number of cases with lipocalin-2 (LCN2) and matrix metalloproteinase-9 (MMP9) measurements and subsequently, the number of cases with matched values for fold-change from baseline ratios available for down-stream analyses.

**Table S2** Progression-free survival (PFS) – univariate analysis

|  |  | HR | 95% CI | *p*-value |
| --- | --- | --- | --- | --- |
| Post-NACT | ADIPOQ | 2.2 | 0.63–7.5 | 0.22 |
|  | ANG | 3.8 | 0.68–21 | 0.13 |
|  | IL6ST | 0.81 | 0.08–8.1 | 0.86 |
|  | TNFRSF11B | 1.17 | 0.45–3.0 | 0.75 |
| Post-CRT | ADIPOQ | 0.79 | 0.24–2.6 | 0.69 |
|  | ANG | 0.42 | 0.07–2.4 | 0.33 |
|  | IL6ST | 0.41 | 0.07–2.5 | 0.33 |
|  | TNFRSF11B | 0.69 | 0.30–1.6 | 0.37 |

Association between altered serum values (array fluorescence intensities as fold-change from baseline) of the four listed proteins (identities given by the gene names) at completion of induction neoadjuvant chemotherapy (post-NACT) and sequential chemoradiotherapy (post-CRT), entered as continuous data from lowest to highest measurements, and PFS was modeled by Cox regression analysis and given as adjusted hazard ratio (HR) with 95% confidence interval (CI); HR above 1: higher probability of unfavorable PFS for the higher value(s) of the parameter. In neither case, altered array values at completion of NACT or CRT were associated with PFS.

**Table S3** Study patients’ characteristics

|  | |  | **Total** |  | **LCN2** | |  | **MMP9** | |
| --- | --- | --- | --- | --- | --- | --- | --- | --- | --- |
|  | |  | **(*n* = 66)** |  | **Post-NACT (*n* = 50)** | **Post-CRT (*n* = 48)** |  | **Post-NACT (*n* = 61)** | **Post-CRT (*n* = 57)** |
|  | |  | *n* (%) |  | *n* (%) | *n* (%) |  | *n* (%) | *n* (%) |
| Median age,  years (range) | | | 58 (30–73) |  | 58 (30–73) | 58 (30–73) |  | 58 (30–73) | 57 (30–73) |
| Sex | Female | | 26 (39) |  | 21 (42) | 17 (35) |  | 25 (41) | 21 (37) |
|  | Male | | 40 (61) |  | 29 (58) | 31 (65) |  | 36 (59) | 36 (63) |
| TN stage | T2–3 | | 44 (67) |  | 36 (72) | 30 (63) |  | 43 (70) | 38 (67) |
|  | T4 | | 22 (33) |  | 14 (28) | 18 (38) |  | 18 (30) | 19 (33) |
|  | N0–1 | | 17 (26) |  | 13 (26) | 14 (29) |  | 16 (27) | 15 (27) |
|  | N2 | | 48 (74) |  | 37 (74) | 34 (71) |  | 44 (73) | 41 (73) |
|  | ND | | 1 |  | 0 | 0 |  | 1 | 1 |
| ypTN stage | ypT0–2 | | 37 (57) |  | 29 (59) | 25 (53) |  | 36 (60) | 33 (59) |
|  | ypT3–4 | | 28 (43) |  | 20 (41) | 22 (47) |  | 24 (40) | 23 (41) |
|  | ND | | 1 |  | 1 | 1 |  | 1 | 1 |
|  | ypN0 | | 47(72) |  | 36 (73) | 35 (74) |  | 43 (72) | 41 (73) |
|  | ypN1–2 | | 18 (28) |  | 13 (27) | 12 (26) |  | 17 (28) | 15 (27) |
|  | ND | | 1 |  | 1 | 1 |  | 1 | 1 |
| TRG score | TRG1–2 | | 47(72) |  | 34 (69) | 31 (66) |  | 42 (70) | 40 (71) |
|  | TRG3–5 | | 18 (28) |  | 15 (31) | 16 (34) |  | 18 (30) | 16 (29) |
|  | ND | | 1 |  | 1 | 1 |  | 1 | 1 |

The initial population of 66 study patients had serum sampled at baseline, following induction neoadjuvant chemotherapy (post-NACT) and sequential chemoradiotherapy (post-CRT), and at evaluation of the neoadjuvant treatment. One patient had disease progression in the pelvic cavity during the neoadjuvant treatment and therefore proceeded to palliative surgery. As a consequence, histologic tumor response data (ypTN stage and tumor regression grade (TRG) scoring) were missing.

The serum samples were analyzed with an antibody array. Array data processing led to reduction in the number of cases with lipocalin-2 (LCN2) and matrix metalloproteinase-9 (MMP9) measurements and subsequently, the number of cases with matched values for fold-change from baseline ratios – given here – that were available for down-stream analyses.

Distribution of parameters was analyzed using Pearson product correlation for continuous data and paired *t*-test for categorical variables. Within the total study population as well as all of the LCN2 and MMP9 groups of study cases, patients’ characteristics were equally distributed.

**Table S4** Treatment-induced enteropathy in study patients

| **Post-NACT serum values** | Group | *n* (%) | Mean (standard deviation) | *p*-value |
| --- | --- | --- | --- | --- |
| LCN2 | 0 | 16 (40) | 0.55 (0.33) |  |
|  | 1 | 10 (25) | 0.50 (0.28) |  |
|  | 2 | 9 (23) | 0.64 (0.66) |  |
|  | 3 | 5 (12) | 0.89 (0.53) |  |
|  | Total | 40 (100) | 0.62 (0.41) | 0.28 |
| MMP9 | 0 | 17 (35) | 0.57 (0.40) |  |
|  | 1 | 17 (35) | 0.77 (0.81) |  |
|  | 2 | 10 (20) | 0.63 (0.71) |  |
|  | 3 | 5 (10) | 0.74 (0.49) |  |
|  | Total | 49 (100) | 0.67 (0.60) | 0.62 |
|  |  |  |  |  |
|  |  |  |  |  |
| **Post-CRT serum values** | Group | *n* (%) | Mean (standard deviation) | *p*-value |
| LCN2 | 0 | 18 (47) | 0.72 (0.42) |  |
|  | 1 | 11 (29) | 0.65 (0.47) |  |
|  | 2 | 6 (16) | 0.57 (0.24) |  |
|  | 3 | 3 (8) | 0.85 (0.40) |  |
|  | Total | 38 (100) | 0.79 (0.58) | 0.75 |
| MMP9 | 0 | 19 (40) | 0.70 (0.66) |  |
|  | 1 | 18 (37) | 1.1 (2.0) |  |
|  | 2 | 7 (15) | 0.66 (0.33) |  |
|  | 3 | 4 (8) | 0.69 (0.43) |  |
|  | Total | 48 (100) | 0.82 (1.1) | 0.92 |

Using one-way analysis of ranks, altered serum values (array fluorescence intensities as fold-change from baseline) of lipocalin-2 (LCN2) and matrix metalloproteinase-9 (MMP9) following induction neoadjuvant chemotherapy (post-NACT) and sequential chemoradiotherapy (post-CRT) were compared for patients categorized into four groups of maximum diarrhea scores, as assessed by Common Terminology Criteria for Adverse Events (CTCAE) version 3.0, during the neoadjuvant treatment course. Categories are Group 0: CTCAE grade 0 diarrhea; Group 1: CTCAE grade 1 diarrhea; Group 2: CTCAE grade 2 diarrhea; Group 3: CTCAE grade 3 diarrhea. No correlations were found between treatment-induced alterations in array values of LCN2 or MMP9 and diarrhea scores.
