# Supplementary material for: Circulating proteins in response to combined-modality therapy in rectal cancer identified by antibody array screening
Source: BMC Cancer. 2016 Jul 26;16:536. doi: 10.1186/s12885-016-2601-x (PMC4962367; doi:10.1186/s12885-016-2601-x)
Supplement: Additional file 2: Figure S1. — Alterations in serum levels of lipocalin-2 (LCN2; left panel) and matrix metalloproteinase-9 (MMP9; right panel) – progression-free survival (PFS). Patients were categorized into groups according to the magnitude of decline in serum values (array fluorescence intensities as fold-change from baseline) at completion of induction neoadjuvant chemotherapy which resulted in a value either below (solid line) or above (dashed line) a calculated optimum threshold (by receiver-operating characteristic analysis) for favorable PFS. Crude differences in PFS were assessed by the log-rank test and visualized by the Kaplan-Meier method. Patients with fold-change values below threshold (i.e., more decline from baseline) during the chemotherapy had significantly better 5-year PFS than those with fold-change values above threshold (i.e., less decline from baseline); for LCN2: 76% versus 38% (p = 0.004, n = 50, threshold of 0.8); for MMP9: 76% versus 20% (p = 0.0002, n = 61, threshold of 1.0). The corresponding identification of optimum threshold values for LCN2 and MMP9 alterations at completion of the sequential chemoradiotherapy was not possible with the actual data sets. (DOC 1827 kb) [file 12885_2016_2601_MOESM2_ESM.doc]

**Additional file 2**

**Fig. S1** Alterations in serum levels of lipocalin-2 (LCN2; left panel) and matrix metalloproteinase-9 (MMP9; right panel) – progression-free survival (PFS)


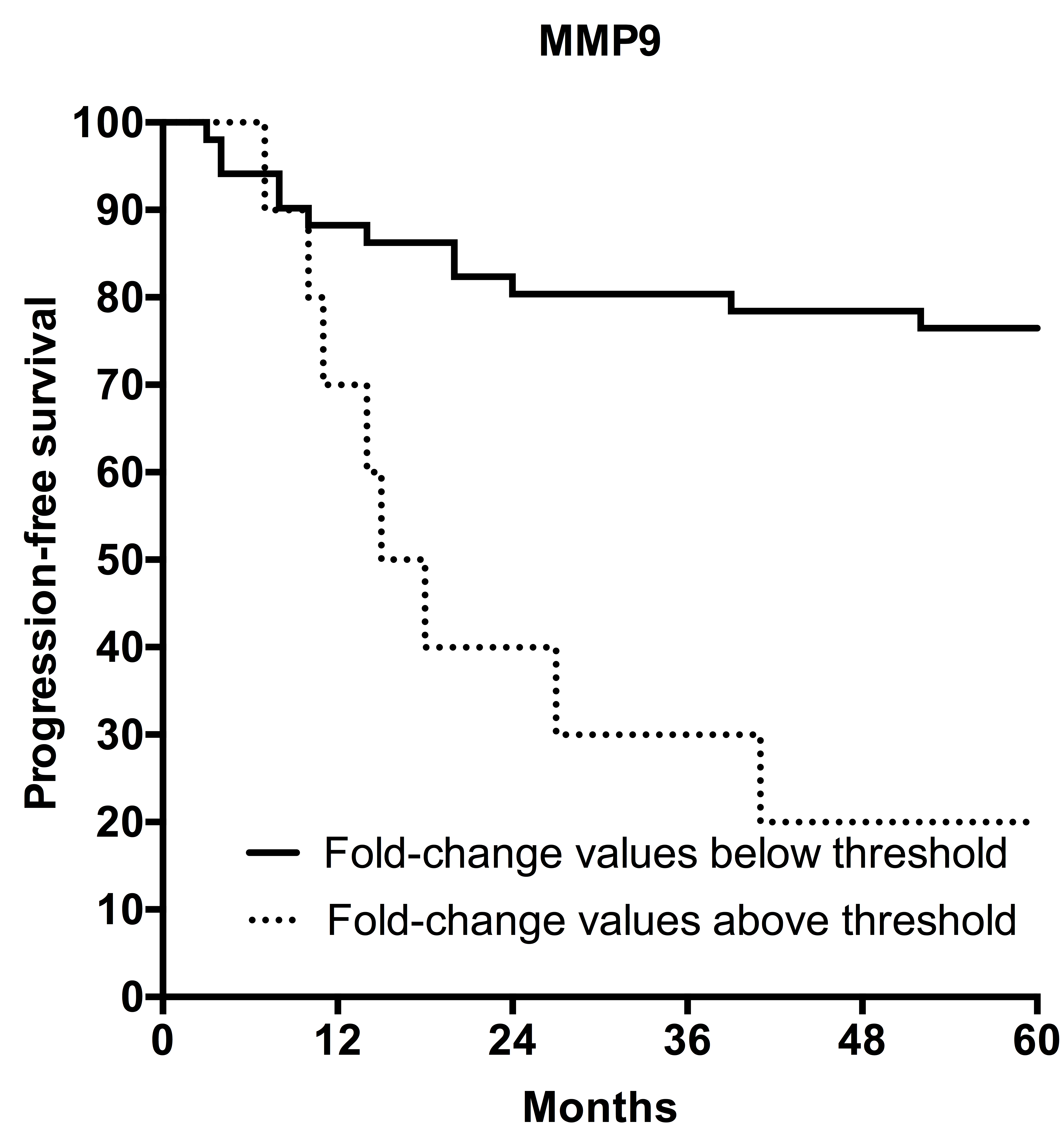

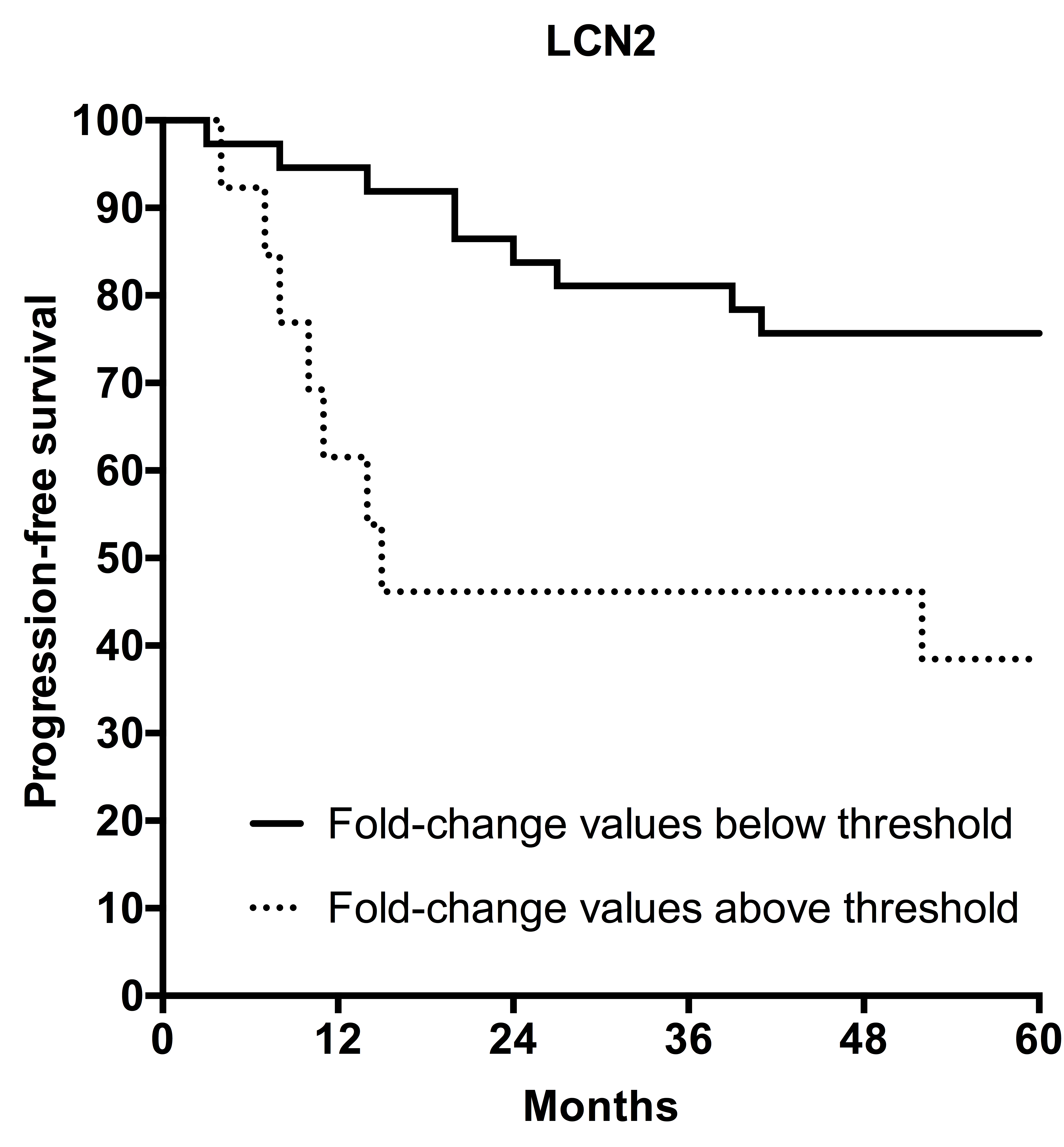


Patients were categorized into groups according to the magnitude of decline in serum values (array fluorescence intensities as fold-change from baseline) at completion of induction neoadjuvant chemotherapy which resulted in a value either below (solid line) or above (dashed line) a calculated optimum threshold (by receiver-operating characteristic analysis) for favorable PFS. Crude differences in PFS were assessed by the log-rank test and visualized by the Kaplan-Meier method.

Patients with fold-change values below threshold (*i.e.,* more decline from baseline) during the chemotherapy had significantly better 5-year PFS than those with fold-change values above threshold (*i.e.,* less decline from baseline); for LCN2: 76% *versus* 38% (*p* = 0.004, *n* = 50, threshold of 0.8); for MMP9: 76% *versus* 20% (*p* = 0.0002, *n* = 61, threshold of 1.0).

The corresponding identification of optimum threshold values for LCN2 and MMP9 alterations at completion of the sequential chemoradiotherapy was not possible within the actual data sets.
